# The HDR CARE Scale, Inpatient Version: A validated survey instrument to measure environmental affordance for nursing tasks in inpatient healthcare settings

**DOI:** 10.1371/journal.pone.0258815

**Published:** 2021-10-20

**Authors:** Renae K. Rich, Francesqca E. Jimenez, Cheryl Bohacek, Alexandra Moore, Abigail J. Heithoff, Deborah M. Conley, Jeri Brittin

**Affiliations:** 1 Research, HDR Architecture, Inc., Omaha, Nebraska, United States of America; 2 Research, HDR Architecture, Inc., Seattle, Washington, United States of America; 3 Oncology, Methodist Hospital, Omaha, Nebraska, United States of America; 4 Geriatrics, Methodist Hospital, Omaha, Nebraska, United States of America; 5 Research, HDR Architecture, Inc., Boise, Idaho, United States of America; Universitat Oberta de Catalunya, SPAIN

## Abstract

Rigorous healthcare design research is critical to inform design decisions that improve human experience. Current limitations in the field include a lack of consistent and valid measures that provide feedback about the role of the built environment in producing desirable outcomes. Research findings about nurses’ efficiency, quality of care, and satisfaction related to inpatient unit designs have been mixed, and there was previously no validated instrument available to quantitatively measure nurses’ ability to work efficiently and effectively in their environment. The objective of this study was to develop, refine, and validate a survey instrument to measure affordance of the care environment to nurse practice, based on various aspects of their work in inpatient units. The HDR Clinical Activities Related to the Environment (CARE) Scale Inpatient Version was developed using item design, refinement, and reliability and validity testing. Psychometric methods from classical test theory and item response theory, along with statistical analyses involving correlations and factor analysis, and thematic summaries of qualitative data were conducted. The four-phase process included (1) an initial pilot study, (2) a content validation survey, (3) cognitive interviews, and (4) a final pilot study. Results from the first three phases of analysis were combined to inform survey scale revisions before the second pilot survey, such as a reduction in the number and rewording of response options, and refinement of scale items. The updated 9-item scale showed excellent internal consistency and improved response distribution and discrimination. The factor analysis revealed a unidimensional measure of nurse practice, as well as potential subscales related to integration, efficiency, and patient care. Within the healthcare design industry, this scale is much needed to generate quantitative and standardized data and will facilitate greater understanding about the aspects of an inpatient healthcare facility that best support nurses’ ability to provide quality patient care.

## Introduction

Rigorous healthcare design research is critical to advancing knowledge to inform design decisions that support desirable human outcomes. Safe, high quality healthcare delivery is a complex adaptive system including interactions of agents at multiple levels [1, 2]. Viewed through the lens of human-environment research, the built environment provides an important context to influence the complex adaptive system of healthcare delivery. Current limitations in the design research field include lack of consistent and valid measures for constructs about the role of the built environment in producing desirable outcomes. While the literature includes various means of measuring the relationships between healthcare personnel [3, 4], between healthcare personnel and the patient care process [5] and between healthcare personnel and the organization [6, 7], no scales specifically measure the relationship between healthcare personnel and the built environment in terms of the delivery of quality inpatient care. Research findings about nurses’ efficiency, ability to provide quality care, and satisfaction related to inpatient unit designs have been mixed, and measurement has been heterogenous. To date, there has been no validated instrument available to quantify nurses’ experience of the patient care environment related to unit design. The important link between the built environment, staff experience, and high-quality patient care needs to be explored and measured.

### Review of the literature

Nursing researchers have developed scales to measure constructs such as collaboration and the related concepts of teamwork and communication. Dougherty and Larson [3] recommended five such scales: the Collaborative Practice Scale (CPS), Collaboration and Satisfaction about Care Decisions (CSACD), ICU Nurse-Physician Questionnaire, Nurses’ Opinion Questionnaire (NOQ), and the Jefferson Scale of Attitudes Toward Physician Nurse Collaboration. These authors went on to develop the Nurse-Nurse Collaboration (NNC) Scale [4]. Liao et al. [8] also developed a Nurse-Nurse Collaboration Behavior Scale to measure specific behaviors associated with nurse peer group relationships and interpersonal interaction within the process of patient-centered care, and Kenaszchuk et al. [9] developed and validated the Interpersonal Collaboration (IPC) scale to assess collaboration between multiple health provider groups, including nurse-nurse and nurse-provider collaboration.

Development of measurement tools that assess nurse perspective of the patient care process has continually evolved [2]. The Nursing Work Index (NWI) [10] was designed to measure nurse activities and practice. Scales derived from the NWI, such as the widely-used Practice Environment [6], measure the structure of the work environments rather than nurse practice. Kramer and Hafner [10] devised the Essentials of Magnetism (EOM) process measurement tool which was later updated [7]. Versions of the EOM measure characteristics that staff nurses at magnet hospitals identified as essential to quality patient care and a satisfying work environment. These authors and other colleagues also tested the Essentials of Professional Nursing Practice (EPNP) scale to assess the extent to which nurses actually engage in these practices [2]. While these instruments consider physical layout and cleanliness of the hospital, as well as availability of equipment and supplies, they do not fully seek to understand the outcomes elicited through human-environment interaction or causal pathways linking the built environment to the delivery of quality care.

Given the ongoing problem of nurse staffing and retention, there have also been studies addressing nurse job satisfaction, burnout, and engagement. A review of instruments measuring job satisfaction in a hospital environment identified seven scales with adequate reliability and validity [11]. Simpson [12] identified the Work Life Model [13], the Job Demands-Resources Model [14], and the Profession Practice Model as useful for measuring employee work engagement [15]. However, given that environmental variables were not included in these models, this author recommended that researchers should conceptualize beyond current models [12].

A majority of studies engaging nurses to assess healthcare design utilize survey methods that are developed by the researchers with little to no testing or validation of the questions. These may ask about the experience or satisfaction with the environment [16–22] or nurse outcomes, such as job satisfaction [23–27], job stress and demands [24, 26, 28, 29], efficiency [25, 27, 29–31], or teamwork and communication [26, 27, 30, 32]. Some studies make use of survey instruments developed and published by other authors, but routine use of previously tested instruments across the healthcare design field is low. The most commonly employed and cited scales are the Maslach Burnout Inventory [23, 33–36], the Nursing Work Index, especially the Practice Environment Subscale [23, 36–39], the Perceived Stress Scale [38, 40], and the Physical Comfort subscale of the Work Environment Scale [16, 41, 42]. The use of nurse surveys in healthcare design research is common, but there is very little consistency and replication of instruments across studies.

### Conceptual model and research aim

**Fig 1:**
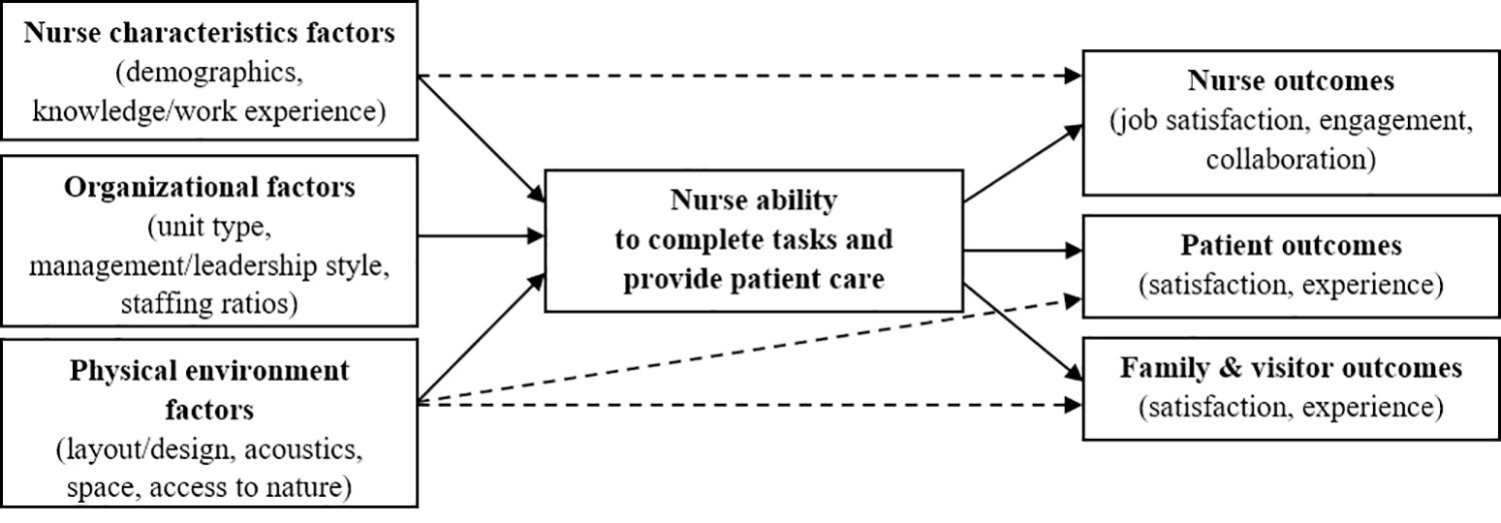
Conceptual diagram of theoretical framework. Hypothesized relationships shown with solid arrows, previously studied direct effects between factors and outcomes as dashed arrows.

While some literature has posited potential direct associations between the factors and outcomes outside of their connection to nursing tasks [13, 26, 43–45], as shown with dashed arrows in Fig 1, it is highly plausible that nurses’ ability to complete tasks related to providing patient care is actually an important mediator in those pathways. Nurse characteristics, organizational factors, and the physical environment may affect nurses’ ability to provide quality patient care, which then affects outcomes for nurses, patients, families, and visitors. These associations are shown in Fig 1 with solid arrows. The means to measure the construct of nurse task affordance is an important step to testing this theory.

This study operationalized an important link between the built environment, nursing staff experience, and high-quality patient care. The specific aim was to develop, refine, and validate a survey instrument to measure nurses’ ability to work efficiently and effectively in their environment, based on routine tasks to care for patients in inpatient units. The construct was intentionally focused on the effort required by nurses to effectively do their jobs rather than asking respondents to attribute the ease of tasks to their physical environment, and as such, relies on the application of affordance in healthcare architecture. Gibson coined the term affordance to describe the possibilities within the environment which affect perception and constrain action [46]. Affordance theory can help explain the interactions between people (i.e. occupants) and the built environment [47]. This scale was developed to specifically assess how well the healthcare built environment supports nurses’ work interactions and task performance. The survey instrument was developed using item design, refinement, and reliability and validity testing. The present study was necessary to ensure the instrument measures what it was intended to measure and can provide a credible and useful tool to healthcare research and design practitioners. The four-phase development and evaluation process resulted in a reliable, valid, and useful survey scale of environmental affordance for inpatient nurse activities, named the HDR Clinical Activities Related to the Environment (CARE) Scale Inpatient Version.

## Materials and methods

### Study design

A mixed-methods, multi-phased approach was used for the evaluation, revision, and validation of the survey instrument. Early survey item development was based on a review of existing post occupancy surveys in healthcare settings, and leaned particularly on the Center for the Built Environment Occupant Toolkit [48, 49]. Survey items underwent further iteration in response to research questions explored, and gaps identified, in the literature examining clinical caregivers’ relationship to the healthcare built environment [50, 51]. The initial survey items were written to assess the key nurse activities and interactions identified in the literature, which included nurse walking fatigue, charting, ability to spend time with patients, and teamwork, especially in emergent situations. The instrument was pilot tested for reliability and validity in the initial and updated stages. Content development included a content validity survey completed by subject matter experts and cognitive interviews with sample participants from the target population. The methodology for the development and evaluation of the survey instrument used best practices from the field of survey research and design, and recommendations in survey development publications by industry experts [52–55].

In the initial pilot survey phase, we assessed the preliminary survey content and performance and documented baseline measures for comparison to repeated measures in the final pilot survey phase. In the content validity survey phase, we collected quantitative and qualitative feedback about the initial survey items from a panel of experts in inpatient nursing and facilities planning. The aim of the cognitive interview phase was to understand the thought process and interpretation of nurses as they completed the survey questions. Finally, following instrument refinement, the final pilot survey phase assessed the updated survey scale content and performance.

### Samples and procedures

The primary study population consisted of nursing staff providing patient care in inpatient hospital units. This includes those in nursing and nurse assistant roles across all types of inpatient care. Subject matter experts in inpatient nursing job types and healthcare built environments were also consulted in the content validity phase of the study.

In the initial pilot study phase, inpatient nursing staff from three hospitals responded to the survey scale in its preliminary state as part of larger studies related to design and construction of new or expanded inpatient hospital facilities. An online survey that included the initial survey instrument was conducted at Great Plains Health (GPH) in May-June 2017, Fremont Health (FH) in June-August 2017, and Parkland Hospital (PH) in June 2018. Respondents in nursing roles who received and answered the initial survey instrument question were included in the scale development study. The survey question asked respondents to specify their level of satisfaction with ten statements representing different nursing tasks (Table 1) using a 7-point response option scale from 1 = *Very dissatisfied* to 7 = *Very satisfied*. In addition to the initial nurse instrument, external survey questions for associated factors and outcomes in the pilot study phases consisted of psychometrically-validated scales or scales that were developed, tested, and refined for content validity by HDR Research based on clarity, relevance, and completeness [52].

**Table 1:** Initial survey instrument used in the initial pilot survey phase.

Question stem	**Please indicate the extent to which you are currently satisfied or dissatisfied which each aspect of your work in your unit.**
Response options	(1) *Very dissatisfied*, (2) *Dissatisfied*, (3) *Somewhat dissatisfied*, (4) *Neither satisfied nor dissatisfied*, (5) *Somewhat satisfied*, (6) *Satisfied*, (7) *Very satisfied*
Scale items	1. My ability to know when my coworkers need help
	2. The amount of time it takes to get help when I need it
	3. The frequency of interactions with my coworkers
	4. The amount I walk during my shift
	5. The amount of time I spend charting
	6. My ability to visually monitor patients without disturbing them
	7. The quality of care that my patients receive
	8. The amount of time spent with my patients
	9. The time it takes me to respond to my patients’ needs
	10. The noise level in patient rooms

In March-April 2019, 167 subject matter experts in nursing and healthcare design were invited to complete the online content validity survey. These included 143 nurse leaders and managers at Nebraska Methodist Hospital Women’s Hospital and 24 clinical or health architectural planners at HDR. Participants were presented with the initial nurse survey instrument and asked to respond to a series of questions about each scale item based on the components of content validity presented by DeVellis [52]. Content validity was evaluated in terms of three attributes: (1) Relevance: how well the items relate to the construct of interest (response options from 1 = *Not at all relevant* to 4 = *Highly relevant*), (2) Clarity: how understandable the items are to survey participants (response options from 1 = *Very unclear* to 4 = *Very clear*), and (3) Completeness: if all important aspects of the construct are covered by the items (response options 1 = *Yes* and 0 = *No*). The construct of interest was stated as “Nurses’ ability to work efficiently and effectively in an inpatient hospital environment” and was further defined as nurse perception of their efficiency, ability to provide quality care, and satisfaction related to the design of the inpatient unit in which they work.

Recruitment for the cognitive interviews was done at Nebraska Methodist Hospital’s main campus (NMH) and Women’s Hospital (MWH), and interviews were conducted at both sites. Inpatient nursing staff from each site were stratified by unit type and randomly selected proportional to strata size to be invited to participate. Participants first completed the online survey instrument on their own, then responded to semi-structured interview questions asked by a team of two researchers—a design researcher from HDR and a nurse researcher from Methodist. Interviews were scheduled for 30 minutes and were conducted in May 2019. Semi-structured interview questions (Table 2) and procedures of the cognitive interviews were based on recommendations for survey evaluation by Fowler [54], DeVellis [52], Dillman et al. [53], and Presser et al. [55], and asked participants to elaborate on the thought process as they completed each survey item, such as how they interpreted the meaning of the statement, whether they had any trouble interpreting it, and the reason behind the response choice they selected. Specific questions also sought to understand the implications of the response options and aspects of nursing that may not have been covered.

**Table 2:** Semi-structured questions for cognitive interviews.

**General item questions**	
	Did you have any trouble understanding the meaning of this item?
	What were you thinking about while answering this item?
	Why did you choose the answer you did for this item?
	Did you consider any aspect of the environment in your response?
**Specific item questions**	
1. Coworkers need help	
	What kind of situations did you think of?
	Did you think about emergency/urgent or non-emergent situations?
2. Time get help	
	What kind of situations did you think of?
	Did you think of this item in the same or a different way as the previous one?
3. Coworker interactions	
	What types of interactions or topics of communication (patient care, social, etc.) did you think of?
	What modes of communication did you consider (face-to-face, electronic)?
	Do you feel that these interactions contribute to whether or not you feel isolated at work?
4. Amount walk	
	Are there any specific problems that contribute to walking?
	Would you prefer to walk less/more if there was a way to achieve that?
	Did you think more about the distances you have to walk or the amount of time spent on your feet?
5. Time charting	
	Did you think about the location(s) where you chart in terms of the time it takes?
	Does the design affect the way or amount that you chart?
	Does the location affect the efficiency of charting?
	Does the charting location matter for patient care?
6. Monitor patients	
	Is ability to visually monitor patients important?
	What type of monitoring did you think of (e.g., vitals, electronic monitoring, physically looking at patients)?
	Would you interpret the item differently without the word “visually” included?
7. Quality patient care	
	How do you define quality care?
	Did you think about medical outcomes or about the non-medical/nurturing aspects of nursing (e.g., listening to patients, holding their hand)?
8. Time with patients	
	What types of activities did you think of?
	Was it more about where you were (i.e., in the patient room) or what you were doing (i.e., interacting with the patient)?
	What aspects of your job affect the time you are able to spend with patients?
9. Time to respond	
	What did you think of as “patients’ needs”? What types of situations? Were they urgent or not?
	What did you think about as how you know your patient needs something?
	What affects the amount of time it takes?
**General scale questions**	
	Are there other aspects of your job/caring for patients that aren’t addressed?
	What parts of your job do you think of as being affected by the design or physical environment? In what ways does the environment help or hinder your work?
	Do you find it difficult to quantify your feelings as satisfied or dissatisfied? Would different response options be better?

Following the analysis of results from the initial three phases, and revisions to the survey instrument based on these results, an online survey that included the revised survey instrument was conducted at NMH and MWH in September and October 2019. Nursing staff at both locations were invited to participate in the survey. In addition to the nurse survey scale, participants responded to demographic questions and other external questions consistent with those asked in the initial pilot phase and used for content validity assessment.

Research protocols that included data collection activities for the first phase of the study (initial pilot survey) were reviewed and deemed exempt by Western Institutional Review Board (IRB) (1-1003733-1, 1-1061894-1). The subsequent phases of the study were approved for continuing review by the Nebraska Methodist Hospital IRB (FWA 00003377). The IRBs waived the need for direct consent for survey participants in all phases, with the assumption that choosing to respond to the survey constituted implied consent. Written informed consent was obtained by all interview subjects prior to participation in the study.

### Analysis

Validity and reliability are the standards by which survey-based scales are evaluated. An instrument is considered reliable if it produces consistent results and valid if it accurately measures what it is intended to measure [54, 56], This implies that results from the instrument will not change unless there is an actual change in what is being measured, and thus, any observed differences based on survey results can be attributed to real differences between respondents [52]. In order to achieve this, respondents should understand survey questions in a way that is also consistent with the meaning that was anticipated by the researchers, and associations with related constructs should hold true [54].

Quantitative data analysis of pilot survey results from the initial and final phases included exploratory factor analyses, tests of significant correlations for associations with other constructs, chi-square tests for differences in results between response groups, and exploration of item distribution and nonresponse. Analyses explored the univariate distribution of responses of each the survey items and bivariate correlations between each pair of items. An overall Cronbach’s alpha value and item-total correlation measured the scale’s reliability and individual contribution of each item [52, 57]. Factor analysis was used to determine the possible number of latent variables represented by the set of items in the scale, and factor rotation, as appropriate, improved factor interpretability [52, 57, 58]. Techniques from Item Response Theory were employed to assess survey item discrimination and response option distribution [52, 57]. Phase 1 survey responses were weighted to account for the difference in sample size between the hospitals and weighted responses were used throughout the multi-site analyses.

In the final pilot study, expected associations of nursing tasks and delivery of patient care with physical environment factors and nurse outcomes, as outlined in the conceptual framework, were evaluated to test the reliability and construct validity of the survey instrument based on item correlations with individual scale items and mean scale scores [52, 57]. CARE Scale items were compared to results from six external scales. Nurse outcomes were measured using four validated and published scales of collaboration effectiveness [59], collaboration experience [59], and job satisfaction (working and interpersonal subscales) [60]. Two scales internally developed to measure efficiency of space layout (e.g., ability to locate needed items, patients, and staff quickly) and space availability (e.g., sufficient space for patients and families, job functions, supplies and equipment, and collaboration) were considered as related environmental aspects.

To measure the content validity of the survey instrument, the relevance, clarity, and completeness of the questions were evaluated. Coded content validity survey responses were assessed for indications of items in the survey instrument where improvements or revisions were warranted, based on identification by at least 15% of respondents, and transcriptions of the cognitive interview responses were assessed qualitatively for indications of problems with the survey instrument among at least 15% of participants [54]. In addition, content validity indices were calculated from the content validity survey responses related to both relevance and clarity of the items and the scale overall. Responses were dichotomized, and relevance responses of 3 = *Moderately relevant* and 4 = *Highly relevant* were considered “More relevant” and clarity responses of 3 = *Somewhat clear* and 4 = *Very clear* were considered “Clear.” An item-level content validity index (I-CVI) equaled the proportion of respondents who rated that item as “More relevant” or “Clear.” Items with an I-CVI greater than 0.79 were determined to be appropriate, between 0.70 and 0.79 needed revision, and less than 0.70 were considered for elimination [61]. The scale-level content validity index (S-CVI) equaled the average of all I-CVIs [62]. With such a large sample of experts, the risk of chance agreement was very low and not calculated. Descriptive results, such as mean, standard deviation, and proportion of specific response options, were also summarized for quantitative data. Qualitative responses were categorized by item and theme and reviewed for consistency across respondents.

SAS v9.4 (SAS Institute, Cary, NC) was used for quantitative and statistical data analysis. Interview responses and open-ended survey comments were managed and analyzed using qualitative data management software NVivo v10 (QSR International Pty Ltd., 2014, Burlington, VT).

## Results

### Phase 1: Initial pilot survey

A total of 444 respondents who were in nursing roles and completed the nurse survey questions of interest were included in this study, 73 from GPH, 48 from FH, and 323 from PH. A majority of respondents had bedside nursing roles (RN/LPN/LVN), with 17% in other roles, including nurse management, advanced practice nurse, or nursing support. A large majority of respondents were employed full-time (89%) and female (90%). Just more than one-third of respondents (35%) said they supervise others. Detailed demographics by site are in Table 3.

**Table 3:** Phase 1 continuous participant demographics by site.

	FH			GPH			PH		
Continuous Variable	N	Mean (SD)	Median (Q1,Q3)	N	Mean (SD)	Median (Q1,Q3)	N	Mean (SD)	Median (Q1,Q3)
Years at Organization^‡^	48	9.38 (10.16)	5.0 (2.0,13.0)	73	4.67 (5.77)	2.0 (1.0,5.0)	323	6.71 (7.33)	4.3 (1.9,8.9)
Hours worked per week^‡^	48	33.46 (10.68)	36.0 (36.0,38.0)	72	36.83 (7.29)	36.0 (36.0,40.0)	322	38.05 (9.09)	36.0 (36.0,40.0)
Age (years) ^‡^	34	36.59 (10.80)	37.0 (27.0,41.0)	60	34.25 (11.43)	29.0 (26.0,42.5)	323	39.28 (11.48)	38.0 (29.0,46.0)
Categorical Variable						FH Frequency (%)	GPH Frequency (%)		PH Frequency (%)
Division									
Behavioral Health (GPH, PH only)						--	5 (6.9)		5 (1.6)
ICU/Critical Care (FH, GPH only)						12 (25.0)	23 (31.5)		--
Surgical & Trauma Services (PH only)						--	--		113 (35.0)
MedSurg/Telemetry (FH, GPH only)						23 (47.9)	28 (38.4)		--
Medicine Services (PH only)						--	--		125 (38.7)
Women, Infant & Children/LDPRN						13 (27.1)	17 (23.3)		80 (24.8)
Job Role**									
Nurse (RN/LPN/LVN)						41 (85.4)	55 (75.3)		273 (84.5)
Nurse Management (Core/Charge/Unit Manager)						0 (0.0)	1 (1.4)		18 (5.6)
Advanced Practice Nurse (Nurse Practitioner)						0 (0.0)	0 (0.0)		24 (7.4)
Nursing Support (CNA/PCA/Care Coordinator)						7 (14.6)	17 (23.3)		8 (2.5)
Years at Organization*									
Less than one year						1 (2.1)	9 (12.3)		48 (14.9)
1–4 years						22 (45.8)	40 (54.8)		136 (42.1)
5–9 years						9 (18.8)	14 (19.2)		67 (20.7)
10–19 years						9 (18.8)	6 (8.2)		49 (15.2)
20–29 years						3 (6.3)	4 (5.5)		18 (5.6)
30 years or more						4 (8.3)	0 (0.0)		5 (1.6)
Supervise others									
No						34 (70.8)	45 (61.6)		206 (65.0)
Yes						14 (29.2)	28 (38.4)		111 (35.0)
Position type**									
Full-time						41 (85.4)	59 (81.9)		294 (91.0)
Part-time						1 (2.1)	4 (5.6)		22 (6.8)
Temporary/PRN/Contract						6 (12.5)	9 (12.5)		7 (2.2)
Hours worked per week**									
0–16 hours						7 (14.6)	2 (2.8)		8 (2.5)
17–32 hours						2 (4.2)	6 (8.3)		24 (7.5)
33–40 hours						34 (70.8)	58 (80.6)		252 (78.3)
41+ hours						5 (10.4)	6 (8.3)		38 (11.8)
Gender									
Female						43 (95.6)	68 (94.4)		287 (88.9)
Male						2 (4.4)	4 (5.6)		36 (11.2)
Age (years)**									
18–24						3 (8.8)	11 (18.3)		18 (5.6)
25–34						12 (35.3)	27 (45.0)		114 (35.3)
35–44						14 (41.2)	8 (13.3)		94 (29.1)
45–54						2 (5.9)	9 (15.0)		49 (15.2)
55–64						3 (8.8)	4 (6.7)		42 (13.0)
65 and older						0 (0.0)	1 (1.7)		6 (1.9)
Race/Ethnicity (PH only)									
American Indian/Alaska Native						--	--		5 (1.6)
Asian						--	--		82 (25.4)
Black/African American						--	--		46 (14.2)
Hispanic/Latino						--	--		39 (12.1)
White/Caucasian						--	--		151 (46.8)

^†^One-way ANOVA F-test of independent means, *p*<0.05.

^‡^One-way ANOVA F-test of independent means, *p*<0.01.

*Chi-square test of independence, *p*<0.05.

**Chi-square test of independence, *p*<0.01.

--Variable or response option not applicable.

CARE Scale item distributions were skewed to the positive response options (Table 4); all items except one (item 2) had at least 50% positive responses and four had 75% positive responses. Mardia’s test for multivariate normality was applied to obtain statistics of skewness (1270.35, *p* < 0.001) and kurtosis (34.25, *p <* 0.001) to measure departure from normality assumptions. The sample was not drawn from a normal distribution [63].

**Table 4:** Phase 1 CARE items response summary.

CARE Scale Items	N	Mean	SD	Median	% Somewhat to Very dissatisfied	% Neither satisfied nor dissatisfied	% Somewhat to Very satisfied
1. Coworkers need help	444	4.28	1.94	5.00	38.46	6.88	54.66
2. Time get help	442	3.98	1.82	4.00	44.10	7.86	48.04
3. Coworker interactions	443	4.89	1.72	6.00	23.62	6.23	70.15
4. Amount walk	442	4.22	1.75	5.00	36.00	13.89	50.11
5. Time charting	441	4.37	1.58	5.00	30.02	17.37	52.60
6. Monitor patients	444	5.31	1.48	6.00	14.13	9.22	76.65
7. Quality patient care	443	5.61	1.22	6.00	9.02	4.10	86.88
8. Time with patients	444	5.23	1.32	6.00	12.21	11.27	76.52
9. Time to respond	441	4.93	1.49	5.00	21.81	9.99	68.21
10. Noise level	440	5.25	1.36	6.00	13.14	9.29	77.57

Note: A value of 4 (on a 1–7 scale) represents the mid-point of the response options.

In phase 1, a maximum of 4 out of 444 responses were missing on any individual scale item (0.9%) and 10 respondents missing at least one item across the entire scale (2.25%). Little’s Missing Completely at Random (MCAR) test statistic was calculated to determine whether data were MCAR. Based on the test statistic (81.2, *p* = 0.0987), the missing data are MCAR [64].

All items were positively inter-correlated (*p* < .001) with estimated correlation coefficients of at least 0.25. Many items had moderate-to-high bivariate correlations with other items, and two (items 1 and 2) had pair-wise correlations greater than 0.80, which could indicate a problem with multicollinearity. The overall reliability coefficient of 0.89 indicated that a high proportion of the variance in the total scores is attributable to a true score value. As shown in Table 5, item-total correlations ranged from 0.41 to 0.79, and item-deleted alpha values slightly lower than the total alpha value indicated that the deletion of a single item would not have a great effect on the alpha coefficient value. However, item-total correlation for one item (the noise level in patient rooms) was considerably lower than all other items and could be considered for item deletion.

**Table 5:** Phase 1 Cronbach’s coefficient alpha and item-total correlations.

CARE Scale			Cronbach’s alpha
			0.8898
Items	N	Item-total Correlation	Item-deleted Alpha
1. Coworkers need help	434	0.7571	0.8692
2. Time get help	434	0.7870	0.8665
3. Coworker interactions	434	0.6566	0.8770
4. Amount walk	434	0.6243	0.8796
5. Time charting	434	0.5541	0.8841
6. Monitor patients	434	0.5224	0.8859
7. Quality patient care	434	0.6337	0.8800
8. Time with patients	434	0.5886	0.8820
9. Time to respond	434	0.7549	0.8707
10. Noise level	434	0.4148	0.8919

Overall and individual Kaiser’s Measure of Sampling Adequacy values between 0.80 and 0.97 indicated that the data were appropriate for factor analysis. With only one eigenvalue greater than one and explaining 88% of the variance, the analysis indicated that a one-factor model might be most appropriate. However, an investigation of the partial residuals and root mean square residuals suggested that more factors may need to be extracted. Two- and three-factor models reduced these values, and thus, were also considered for rotation and theoretical interpretation. The one-factor model reflected the unidimensional aspect of the correlations between all items with loadings ranging from 0.44 to 0.83 and accounting for 87% of the total common variance between the items. The two-factor model reflected the items that relate more to nursing tasks and efficiency, such as integration between coworkers and time spent in different activities, highly loaded on the first factor and items more related to patient care and experience on the second factor (Table 6). The variance explained by each factor was close to even, with the first factor accounting for 56.2% and the second factor accounting for 46.6% of the common variance. The biggest difference in the three-factor compared to the two-factor model was that it separated the first factor from the two-factor model into two different factors for nurse integration and task efficiency (Table 7). The third factor still represented patient care items. The amount of variance explained by each factor was relatively similar, with 42.8% explained by the first factor, 37.4% by the second, and 32.3% by the third. Site responses were also each analyzed separately. While there were slight differences in the site-specific models, there were common themes between them and with the overall model that support the findings in that factor analysis. Factor analyses using polychoric correlations produced very similar factor loadings and the same interpretations.

**Table 6:** Phase 1 varimax rotated two-factor model loadings.

Items		Factor 1	Factor 2
1.	Coworkers need help	0.88851	0.24756
2.	Time get help	0.83195	0.33426
3.	Coworker interactions	0.69275	0.27770
4.	Amount walk	0.57043	0.33527
5.	Time charting	0.42680	0.38380
8.	Time with patients	0.20466	0.75566
7.	Quality patient care	0.29042	0.72453
9.	Time to respond	0.50392	0.64247
6.	Monitor patients	0.30883	0.48279
10.	Noise level	0.17836	0.46711

**Table 7:** Phase 1 varimax rotated three-factor model loadings.

Items		Factor 1	Factor 2	Factor 3
1.	Coworkers need help	0.84534	0.20804	0.34002
3.	Coworker interactions	0.75820	0.28444	0.15750
2.	Time get help	0.68708	0.23016	0.51171
7.	Quality patient care	0.29612	0.85521	0.12040
8.	Time with patients	0.15706	0.66441	0.32233
9.	Time to respond	0.36702	0.52484	0.50683
10.	Noise level	0.12270	0.39991	0.25634
4.	Amount walk	0.39831	0.16894	0.57251
5.	Time charting	0.25532	0.23015	0.53251
6.	Monitor patients	0.14039	0.34042	0.50702

**Fig 2:**
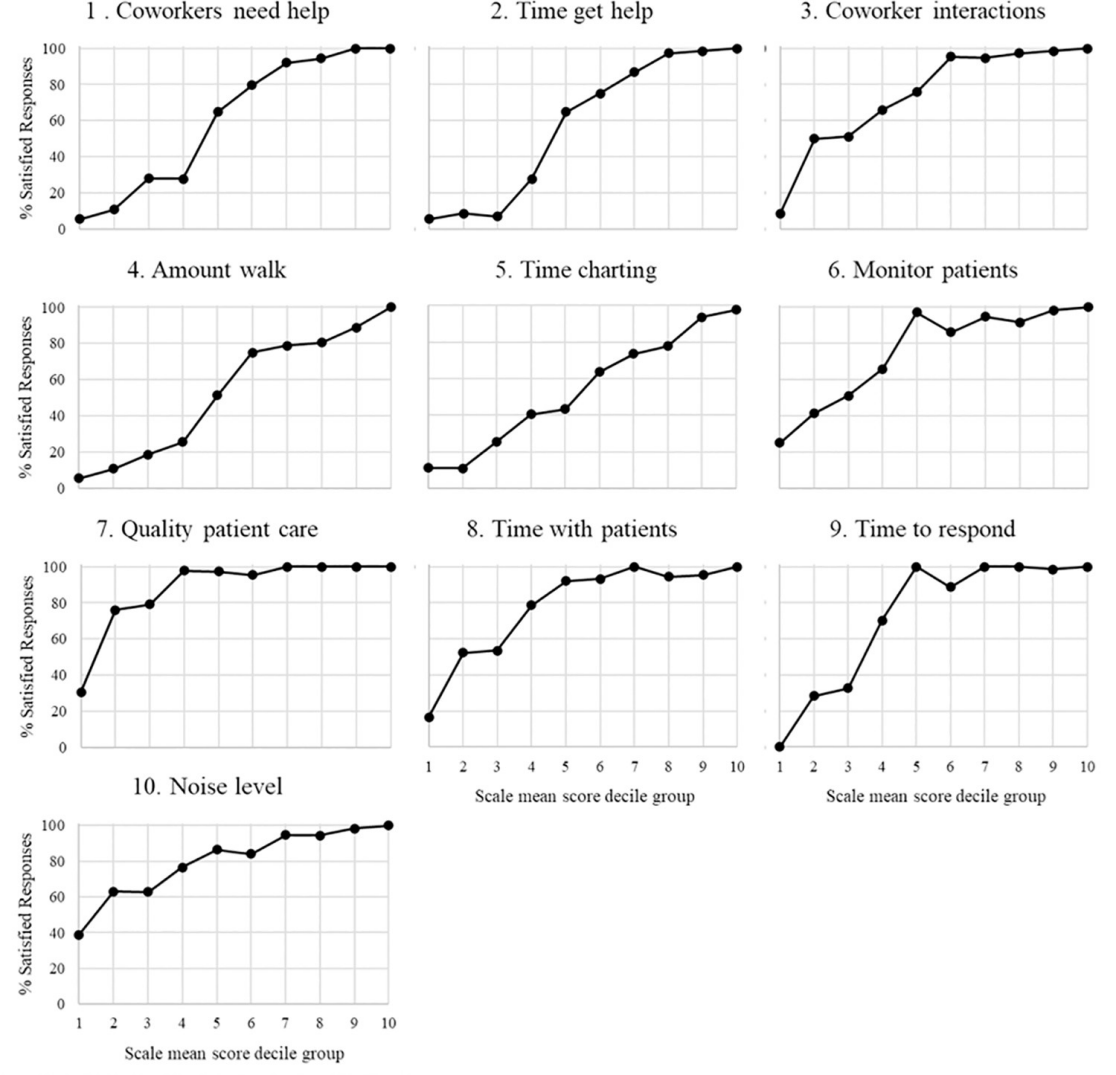
Phase 1 item response curves.

**Fig 3:**
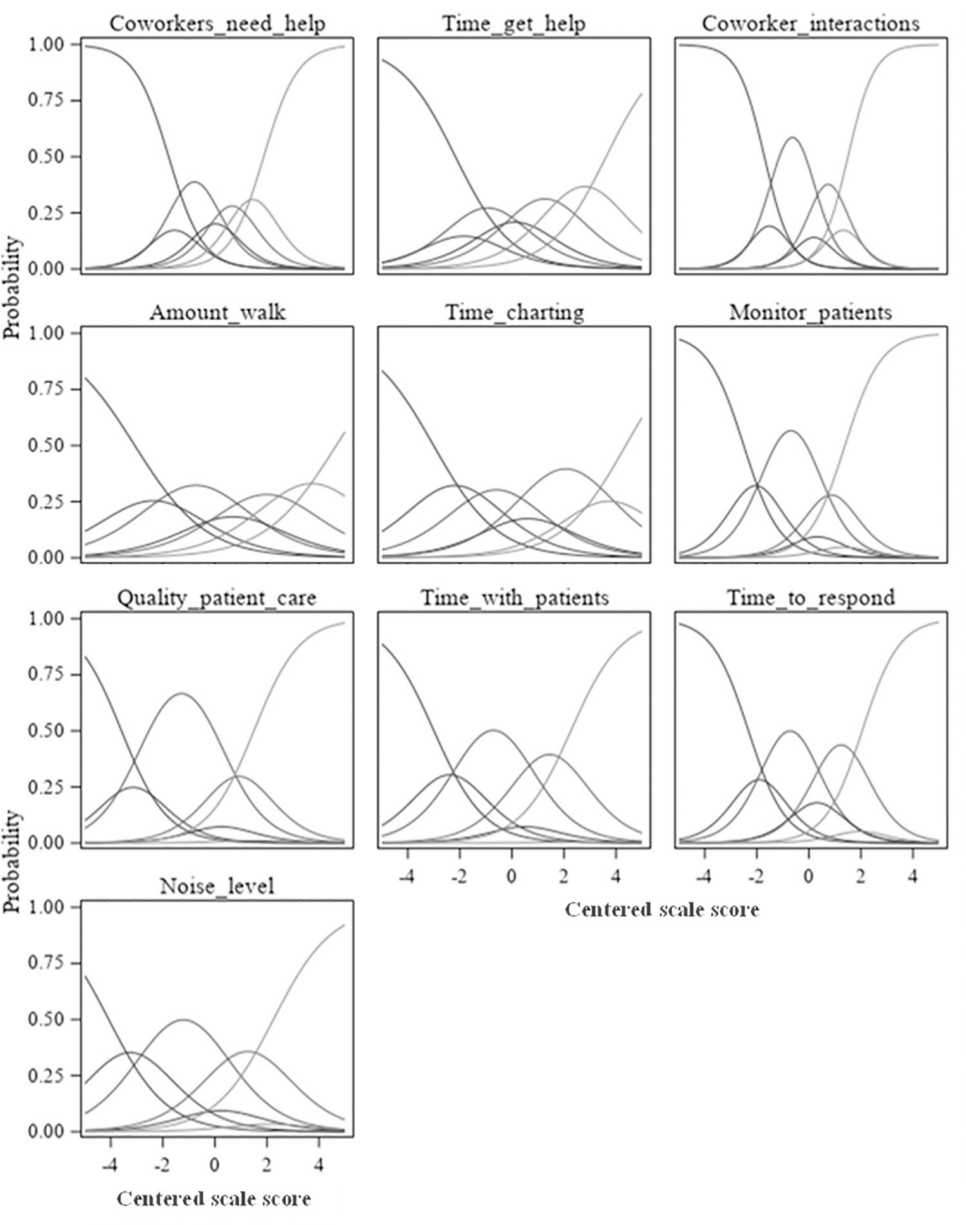
Phase 1 category response curves.

A higher discrimination index is an indication that the item is better able to differentiate between those with the highest and lowest overall scores. Many items had very high discrimination values, with indices ranging from 39.8 to 92.4. The three items (7,8, and 10) with the lowest discrimination indices, between 39.8 and 57.7, were less discriminating among respondents with the lowest overall scores. Similarly, item discrimination curves (Fig 2) showed four items with values above 20% for the lowest CARE Scale mean score decile group, indicating a difficulty in discriminating among lower-scoring respondents. Nearly all items, and five in particular, reached 90% of higher satisfaction well below the highest decile groups. This indicated a need for better discrimination for most items in the upper range of responses, as well. In all category response curves (Fig 3), only three to five response options reached a local maximum across the combined distributions, signifying that there were more response options available to respondents than necessary.

### Phase 2: Content validity survey

A total of 60 respondents completed the content validity survey, 21 from HDR and 39 from Methodist. All HDR respondents have experience in inpatient healthcare facilities planning and design (mean = 18.1 years) and some have experience in other areas, including nursing. All Methodist respondents have experience in nursing (mean = 21.0 years), two-thirds have experience in healthcare administration, and some have experience in healthcare planning or design or research. Nearly all HDR respondents have experience with all types of inpatient units listed (ICU/Critical Care, Med Surg/Acute Care, Progressive Care/Step-Down/Telemetry, Womens & Infants). At least some of Methodist respondents have experience in each inpatient unit type, with the highest proportion in Med Surg/Acute Care (69.2%) and lowest proportion in Womens & Infants (17.9%).

**Fig 4:**
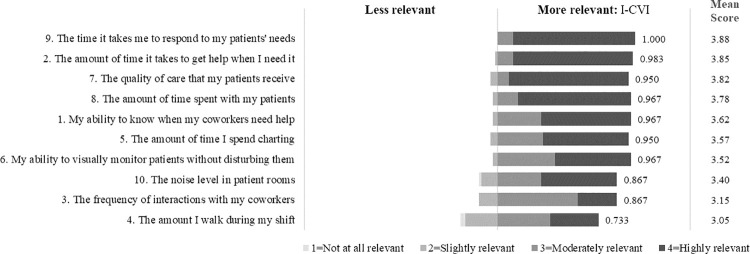
Phase 2 relevance of CARE items response. Frequency, relevance I-CVI, and mean score response.

On a response scale from 1 = *Not at all relevant* to 4 = *Highly relevant*, seven out of ten items had a mean relevance score of 3.5 or higher and all items had a mean score of at least 3.0 (Fig 4). Eight items were rated as moderately or highly relevant by at least 86% of respondents and highly relevant by at least 50%. Nine out of the 10 items had a relevance I-CVI greater than 0.80 (“appropriate”), and item 4 had a I-CVI of 0.733 (“needs revision”). The S-CVI in terms of relevance was 0.925.

**Fig 5:**
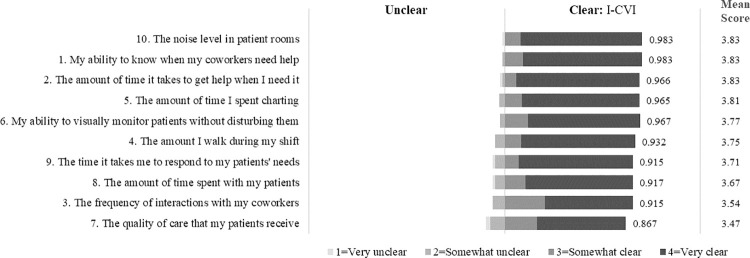
Phase 2 clarity of CARE items response. Frequency, clarity I-CVI, and mean score response.

On a response scale from 1 = *Very unclear* to 4 = *Very clear*, all ten items had a mean clarity score of 3.5 or higher (Fig 5). Eight items were rated as somewhat or very clear by at least 90% of respondents and very clear by at least 75%. All 10 items had a clarity I-CVI greater than 0.80 indicating appropriateness, and the S-CVI in terms of clarity was 0.941. Respondents suggested changes to the item wording that they thought would improve understanding by survey participants. Many thought that the term “quality of care” and types of interactions between coworkers needed to be more specific. One-fourth of respondents thought there was some important aspect of the construct missing from the items; however, there were no consistent suggestions between respondents.

### Phase 3: Cognitive interviews

A total of 44 participants from both Methodist hospitals participated in the cognitive interviews, 61% from NMH and 39% from MWH. Participants were all in a nursing role with an average of 6.8 years at the organization and 9.8 years of hospital experience; a majority were RNs (65.9%) and the remainder were CNAs or charge nurses. All except one unit (9N –Cardiac Critical Care) were represented by at least one participant. The responses to nearly all survey items were more positive than the combined weighted responses in the phase 1 survey. Two items (5 and 6) skewed slightly more towards negative responses among interview participants.

Generally, items were interpreted as intended. However, some nuanced interpretations were evident. For the items related to getting and providing help, most nurses thought of non-emergent situations and a general awareness of what was happening on their unit. The term, “interactions with coworkers” was interpreted differently by nurse respondents and ranged from social interaction, like “chit chat” to conversations centered on patients and their needs to a combination of both professional and social communications. There was a holistic view of quality patient care, and many respondents expressed it in terms of the culture of their organization. When asked how they defined quality of care, most nurses explained that it encompassed the wide range patient experience, from medical outcomes to personalized attention. Most respondents expressed that walking was an expected part of their job and some stated that they enjoyed that their job keeps them physically active. Among nurse respondents, time spent with patients was interpreted to mean one-on-one interaction that usually includes some form of medical or medical-related care such as help with activities of daily living, patient education, or walking, but rarely assessments or checking vitals. The time it takes to respond to patients’ needs was most commonly interpreted to mean response to patient call light. The ability to visually monitor patients without disturbing them was seen as important and was interpreted to mean actually laying eyes on patients, not viewing them through a remote monitoring system. Nurse respondents expressed that they spend a lot of time charting because they cannot chart by exception, and their organization’s policies for charting were seen as having the most impact on the time they spend charting.

When asked if the survey questions missed any important aspect of their jobs, more than half of the respondents said no. However, staffing ratios and patient load were seen by some as an important aspect of the job left out of the questions. Further, design details that could impact patient safety were brought up, for example: phone cords that could be a tripping hazard, the placement of patient bathroom doors and access to the headwall and equipment. Some of these concerns may be specific to the study site. There were mixed responses among nurses as to how they felt about the current survey response options. While some said the response options did not affect their answers, other felt like the satisfaction scale swayed them toward more positive responses. The option of responding on an agree-to-disagree or other type of scale, seemed more objective to some respondents.

### Survey instrument revision

Prior to the final survey phase, results from the first three phases of data collection and analysis were compiled and considered for updates and revisions to the survey instruments. The study team from HDR and Methodist met to review the results to date and discuss the structure and wording of the final survey scale. In order to reduce potential positive bias with satisfaction responses, the updated survey scale asked respondents to specify the amount of effort it typically takes to incorporate different nursing tasks into their work, using a 5-point response option scale from 1 = *Is not possible even with much effort* to 5 = *Occurs naturally without effort* (Table 8). These options more directly relate to the concept of affordance in that the ideal environment should naturally support and facilitate nursing tasks. The question stem and items were also reworded to frame the question around nurses’ work environments and clarify items that respondents found confusing or vague. Due to its lower connection to the other scale items and relevance to the construct, item 10 (*noise level in patient rooms*) was eliminated from the scale entirely.

**Table 8:** Revised CARE scale survey instrument.

Question stem	**In answering the following question, please consider the work environment on your unit. How much effort does it typically take to incorporate each of the following into your work?**
Response options	(1) *Is not possible*, *even with much effort*, (2) *Requires much effort*, (3) *Requires moderate effort*, (4) *Requires little effort*, (5) *Occurs naturally without effort*
Scale items	1. Know what is happening with my coworkers during my shift
	2. Get help as soon as I need it
	3. Frequently interact with my coworkers during my shift
	4. Move throughout my unit in an efficient way (i.e., without backtracking, manageable distances between locations)
	5. Integrate charting into my normal workflow
	6. Visually monitor patients without disturbing them
	7. Provide high quality care
	8. Spend enough time with my patients
	9. Efficiently respond to patient needs

### Phase 4: Final pilot survey

In the final phase, a total of 357 respondents completed the survey containing the updated CARE Scale, 252 from NMH, and 105 from MWH. A majority of respondents were RNs (63.6%), 23.8% were CNAs, and the remaining 12.6% were in other roles, including nurse management, advanced practice nurse, or nursing support. A large majority of respondents were employed full-time (72.6%) and female (94.3%). One-fourth of respondents (24.8%) said they supervise others. Detailed demographics by site are in Table 9.

**Table 9:** Phase 4 participant demographics by site.

	NMH			MWH		
Continuous Variable	N	Mean (SD)	Median (Q1,Q3)	N	Mean (SD)	Median (Q1,Q3)
Years at organization	252	7.44 (9.86)	4.0 (1.0, 9.0)	103	7.27 (9.48)	4.0 (1.0, 9.0)
Years hospital experience^‡^	252	9.10 (10.27)	5.0 (2.0, 12.0)	103	13.13 (13.21)	7.0 (3.0, 19.0)
Hours worked per week^†^	243	33.01 (7.36)	36.0 (25.0, 36.0)	102	30.80 (7.47)	36.0 (24.0, 36.0)
Age (years) ^†^	232	34.10 (11.88)	30.0 (25.0, 39.5)	100	37.60 (13.08)	34.0 (27.0, 43.5)
Categorical Variable			NMH Frequency (%)		MWH Frequency (%)	
Unit						
Acute Rehab			13 (5.16)		--	
Short Stay			20 (7.94)		--	
Oncology			35 (13.89)		--	
Acute Care for Elders			37 (14.68)		--	
Medical/Surgical			27 (10.71)		--	
Cardiac			20 (7.94)		--	
Progressive Care Unit			27 (10.71)		--	
Critical Care Unit			35 (13.89)		--	
Orthopedic / Neurology			21 (8.33)		--	
Cardiac Critical Care			15 (5.95)		--	
GYN/HROB–Overflow Mother Baby			--		13 (12.38)	
Mother Baby			--		22 (20.95)	
NICU			--		49 (46.67)	
Labor & Delivery			--		20 (19.05)	
Other–Multiple Units			2 (0.79)		1 (0.95)	
Job Role**						
CNA–Certified Nursing Assistant			72 (28.57)		13 (12.38)	
Core/Charge Nurse			24 (9.52)		7 (6.67)	
CPA–Certified Patient Assistant			2 (0.79)		0 (0.00)	
Manager/Director			6 (2.38)		2 (1.90)	
NP–Nurse Practitioner			0 (0.00)		4 (3.81)	
RN–Registered Nurse			148 (58.73)		79 (75.24)	
Supervise others*						
No			171 (71.85)		81 (83.51)	
Yes			67 (28.15)		16 (16.49)	
Position type**						
Full-time			195 (77.38)		64 (60.95)	
Part-time			41 (16.27)		30 (28.57)	
Part-time/Per Diem			16 (6.35)		11 (10.48)	
Typical Shift**						
Weekdays			18 (7.14)		2 (1.92)	
Day shift			120 (47.62)		54 (51.92)	
Night shift			106 (42.06)		37 (35.58)	
Evening shift			1 (0.40)		0 (0.00)	
Rotating/varying shifts			7 (2.78)		11 (10.58)	
Gender*						
Female			230 (92.74)		102 (98.08)	
Male			18 (7.26)		2 (1.92)	
Race/Ethnicity						
American Indian/Alaska Native			1 (0.41)		0 (0.00)	
Asian			3 (1.22)		0 (0.00)	
Black/African American			1 (0.41)		0 (0.00)	
Hispanic/Latino			6 (2.44)		3 (2.94)	
Native Hawaiian/Other Pacific Island			0 (0.00)		1 (0.98)	
White/Caucasian			229 (93.09)		96 (94.12)	
Two or more races			6 (2.44)		2 (1.96)	

^†^One-way ANOVA F-test of independent means, *p*<0.05.

^‡^One-way ANOVA F-test of independent means, *p*<0.01.

*Chi-square test of independence, *p*<0.05.

**Chi-square test of independence, *p*<0.01.

—Variable or response option not applicable.

While distributions were still slightly skewed to the positive response options (Table 10), all except one item (regarding charting) had a more centralized distribution than in the cognitive interview survey responses. Mardia’s test for multivariate normality was applied and statistics of skewness (388.99, *p* < 0.001) and kurtosis (9.63, *p* < 0.001) were obtained. The sample was not drawn from a normal distribution [63].

**Table 10:** Phase 4 CARE items response summary.

CARE Scale Items	N	Mean	SD	Median	% Not possible or requires much effort	% Requires moderate effort	% Occurs naturally or requires little effort
1. Know what is happening	356	3.46	1.0	4.0	18.82	29.78	51.40
2. Get help as needed	356	3.79	0.80	4.0	7.02	22.75	70.22
3. Interact with coworkers	355	4.00	0.89	4.0	5.07	23.10	71.83
4. Move in efficient way	357	3.69	0.94	4.0	10.36	29.41	60.22
5. Integrate charting	355	3.62	0.97	4.0	13.80	30.70	55.49
6. Monitor patients	356	3.58	1.05	4.0	16.85	23.31	59.83
7. Provide quality care	355	3.98	0.99	4.0	9.30	20.00	70.70
8. Enough time with patients	354	3.41	1.10	3.0	22.60	29.10	48.31
9. Respond patient needs	355	3.82	0.92	4.0	8.45	25.35	66.20

Note: A value of 4 (on a 1–7 scale) represents the mid-point of the response options.

In the phase 4 dataset, there was a maximum item nonresponse of 3 out of 357 respondents (0.8%) and 5 respondents missing one or more items across the entire scale (1.4%). Item missingness was assessed using Little’s MCAR test (81.2, *p* = 0.099). Based on the results of this testing, missing data for phase 4 are MCAR [64].

All items were positively intercorrelated with estimated correlation coefficients of at least 0.33, and four out of nine items correlated with at least four other items at a 0.50 level or higher. Although a group of three items related to direct patient care were very highly correlated with each other (items 7, 8, and 9), with a maximum pair-wise correlation of 0.75, no correlations indicated a problem with multicollinearity. The overall reliability coefficient value of 0.89 indicated that a high proportion of the variance in the total scores is attributable to a true score value. Item-total correlations (Table 11) ranged from 0.53 to 0.75 and nearly consistent item-deleted alpha values indicated that the deletion of a single item would not have a great effect on the alpha coefficient value.

**Table 11:** Phase 4 Cronbach’s coefficient alpha and item-total correlations.

CARE Scale			Cronbach’s alpha
			0.8925
Items	N	Item-total Correlation	Item-deleted Alpha
1. Know what is happening	352	0.6247	0.8828
2. Get help as needed	352	0.6506	0.8814
3. Interact with coworkers	352	0.6212	0.8830
4. Move in efficient way	352	0.5915	0.8852
5. Integrate charting	352	0.6695	0.8791
6. Monitor patients	352	0.5320	0.8909
7. Provide quality care	352	0.7095	0.8758
8. Enough time with patients	352	0.7316	0.8738
9. Respond patient needs	352	0.7529	0.8728

Overall and individual Kaiser’s Measure of Sampling Adequacy values between 0.84 and 0.94 indicated that the data were very appropriate for a factor analysis. With only one eigenvalue greater than one and explaining 95% of the variance, the analysis indicated that a one-factor model might be most appropriate and the scale might be unidimensional. However, an investigation of the partial residuals and root mean square suggested that more factors may need to be extracted. Two- and three-factor models reduced these values, and thus, were also considered for rotation and theoretical interpretation. The one-factor model reflected the unidimensional aspect of the correlations between all items with loadings ranging from 0.56 to 0.81 and accounting for 94.4% of the total common variance between the items. The two-factor model reflected the items that related more to direct patient care highly loaded on the first factor and items more related to nurse integration and efficiency on the second factor (Table 12). Slightly more variance was explained by the first factor than the second factor, accounting for 64.4% and 46.6%, respectively. The three-factor model separated the lowest-loading items in each of the two factors into a separate third factor, creating three factors representing direct patient care, nurse integration, and task efficiency (Table 13). The first factor accounted for a majority of the variance explained, with 55.5% explained by the first factor, 38.0% by the second, and 26.3% by the third. Factor analyses using polychoric correlations produced very similar factor loadings and the same interpretations.

**Table 12:** Phase 4 varimax rotated two-factor model loadings.

Items		Factor 1	Factor 2
9.	Respond patient needs	0.83630	0.27026
8.	Enough time with patients	0.79899	0.27997
7.	Provide quality care	0.75198	0.28838
5.	Integrate charting	0.63099	0.34198
6.	Monitor patients	0.43359	0.33883
1.	Know what is happening	0.23553	0.83155
3.	Interact with coworkers	0.27880	0.73691
2.	Get help as needed	0.46181	0.52072
4.	Move in efficient way	0.41726	0.45560

**Table 13:** Phase 4 varimax rotated three-factor model loadings.

Items		Factor 1	Factor 2	Factor 3
9.	Respond patient needs	0.82591	0.23880	0.23040
8.	Enough time with patients	0.79150	0.25058	0.21975
7.	Provide quality care	0.72180	0.24750	0.25727
5.	Integrate charting	0.57581	0.26738	0.33010
1.	Know what is happening	0.21968	0.81768	0.24035
3.	Interact with coworkers	0.27159	0.71984	0.21411
2.	Get help as needed	0.42967	0.46890	0.27496
4.	Move in efficient way	0.23828	0.27719	0.73565
6.	Monitor patients	0.32848	0.20068	0.48138

**Fig 6:**
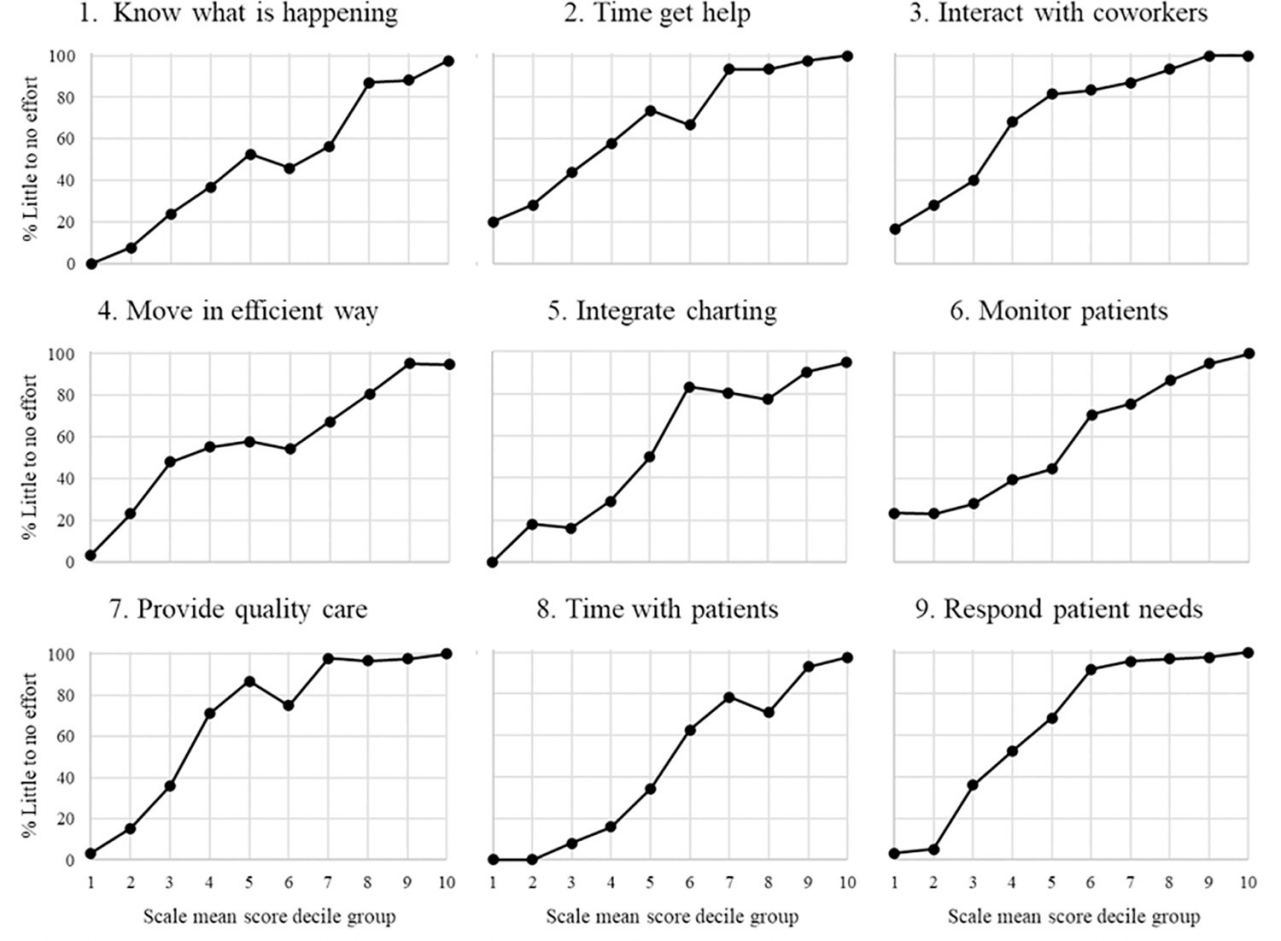
Phase 4 item response curves.

**Fig 7:**
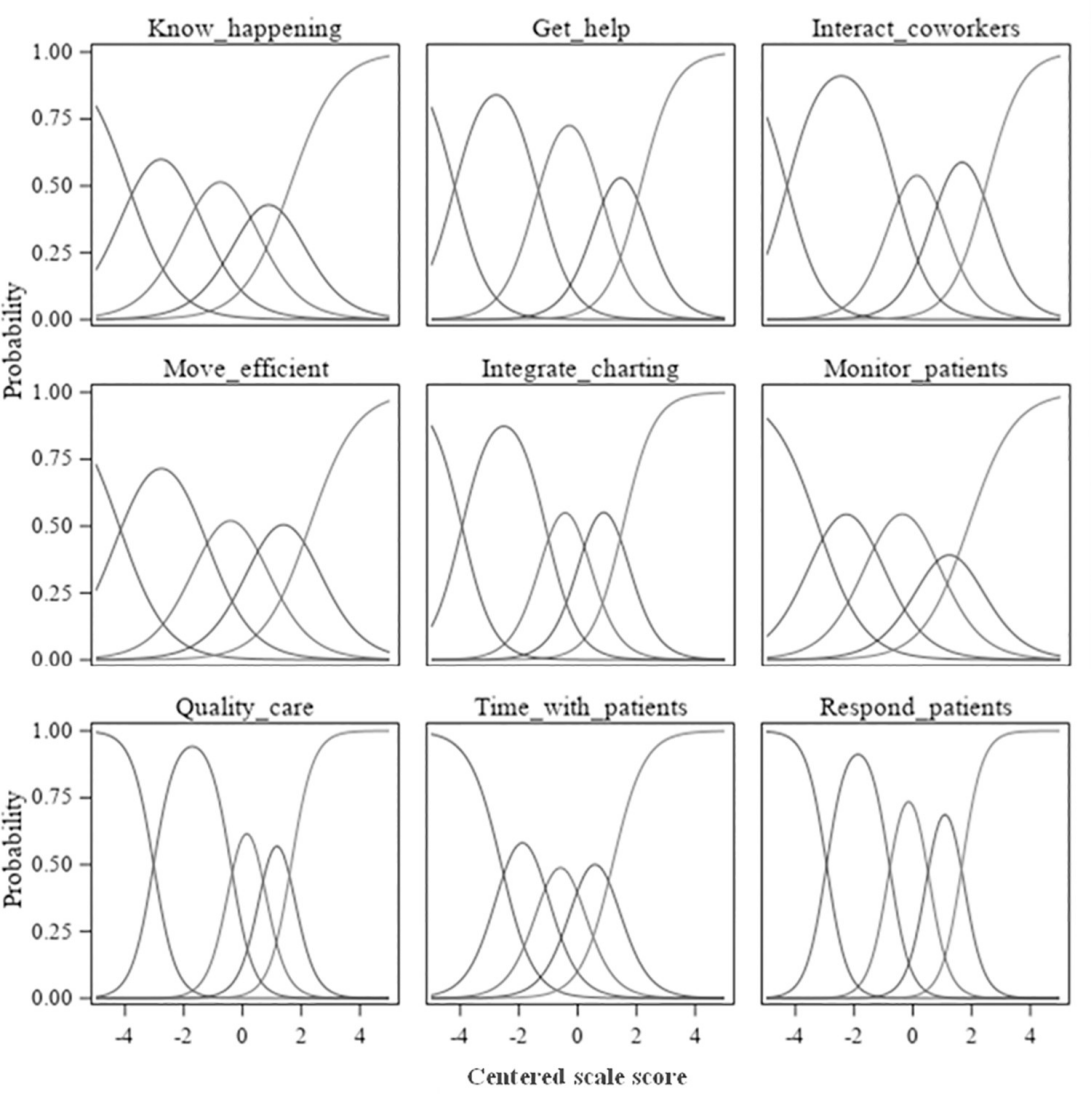
Phase 4 category response curves.

All items had very high discrimination values (indices ranging from 71.4 to 92.6), with much higher proportions of responses in the highest categories (4 = *Requires little effort* and 5 = *Occurs naturally without effort*) among those with scores in the CARE Scale mean score upper quartile compared to those with scores in the lower quartile. In considering the item discrimination curves (Fig 6), only two items had proportions of highest category responses at or above 20% for the lowest CARE Scale mean score decile group and no items had proportions above 25% for this group, indicating good discrimination among lower-scoring respondents. Three items reached 90% or higher proportions by the 6th decile group, and four items reached 90% by the 7th decile group. These items do not discriminate as well among the upper ranges of responses. In all category response curves for all items (Fig 7), all five response options reached a local maximum across the combined distributions and in the logical order, indicating that there are an appropriate number of response options that are ordered as expected.

All external scale scores and nearly all items were significantly positively correlated with all CARE Scale items (Table 14). Collaboration experience was most highly correlated with nurses’ ability to get help as soon as needed (estimated correlation coefficient: *r* = 0.53), followed by ability to know what is happening with coworkers (*r* = 0.39), efficiently responding to patient needs (*r* = 0.39), frequently interacting with coworkers (*r* = 0.37), and spending enough time with patients (*r* = 0.37). Collaboration effectiveness and interpersonal job satisfaction were similar and also most highly correlated with getting help as soon as needed (*r* = 0.35 and *r* = 0.43, respectively). Work job satisfaction was significantly correlated with all CARE items and more similar to interpersonal correlations among items related to patient care and interaction. Wayfinding was most highly correlated to ability to move throughout the unit in an efficient way (*r* = 0.47), but also in response time to patient needs (*r* = 0.36), integration of charting into workflow (*r* = 0.35), and ability to visually monitor patients (*r* = 0.35). Correlation estimates for the CARE items of space availability especially related to ability to integrate charting in workflow (*r* = 0.42) and spend enough time with patients (*r* = 0.41), followed by efficiency of moving throughout the unit (*r* = 0.39), responding to patient needs (*r* = 0.37), and getting help when needed (*r* = 0.37) were all highly significant (*p* < 0.001).

**Table 14:** CARE scale item correlation with external scales.

Alternate scales	CARE Scale Item								
	Know	Help	Interact	Move	Chart	Monitor	Quality	Time	Respond
Collaboration Experience	0.39**	0.53**	0.37**	0.35**	0.33**	0.28**	0.33**	0.37**	0.39**
Collaboration Effectiveness	0.21**	0.35**	0.17**	0.17**	0.18**	0.07	0.24**	0.22**	0.24**
Job Satisfaction—Work	0.25**	0.33**	0.18**	0.21**	0.26**	0.19**	0.30**	0.32**	0.29**
Job Satisfaction—Interpersonal	0.28**	0.43**	0.25**	0.22**	0.30**	0.19**	0.26**	0.33**	0.30**
Efficiency of Space Layout	0.32**	0.33**	0.25**	0.47**	0.35**	0.35**	0.31**	0.28**	0.36**
Space Availability	0.31**	0.37**	0.24**	0.39**	0.42**	0.34**	0.34**	0.41**	0.37**

** Pearson’s correlation coefficient, *p* < 0.01

## Discussion

The results of this study have a direct potential impact on the practice of healthcare design and nursing in that the ability to quantitatively measure nurse experience with job and patient care tasks will provide a missing link to developing clear associations between the factors that influence these tasks, including the physical environment they work in, and the connections to important employee outcomes such as job satisfaction, absenteeism, engagement, and collaboration. Patient experience, often measured through surveys such as the Hospital Consumer Assessment of Healthcare Providers and Systems (HCAHPS), may also be linked to nurse ability to effectively provide care. When planning a new healthcare environment, these outcomes are often found in the list of goals and guiding principles. The HDR CARE Scale Inpatient Version will provide a critical link to understanding how the physical environment can influence these key outcomes. Future research conducted using the scale will feed into the cycle of evidence-based design by generating this knowledge and affecting the design strategies that are implemented.

The updated scale response wording in Table 8 was specifically chosen to reflect the focus of environmental affordance for nurse tasks, asking about the degree to which nurses are able to complete their work without hinderance. The scale items themselves do not ask about the physical environment, but rather focus on nursing tasks, only mentioning the environment in the question stem and framing of the question. This was done by intention, to allow researchers to reach causal inferences on the role of the environment through analyses and associations within and between subjects based on aspects of their surroundings, rather than asking respondents to draw these conclusions themselves. The CARE Scale is intended to measure the nurse experience based on the extent of effort required to complete tasks, but alone not meant to explain the reasons for differences in responses.

Although strong in many aspects even in the earlier phases, the performance of the revised survey scale improved upon the initial scale testing. With high intercorrelations and Cronbach’s alpha, the scale displayed high reliability. Significant correlations in expected directions and intensities with several external scales related to collaboration, job satisfaction, layout, and space adequacy, confirmed the validity of scale questions, originally tested and refined based on the results from the content validity and cognitive interview data collection phases. The updated scale also showed improved and satisfactory performance in terms of item discrimination and response.

A limitation of this study was the diversity of the sample of nurses and locations measured. As the hospital environment is common across those who work on the same unit and even between different units in the same hospital, it will be important to continue to test this instrument in diverse settings with a wide range of respondents. It is expected that a more expansive and varied sample will not negate the results found in this study, but will reveal a better distribution of performance and capabilities. From the factor analysis results, it was concluded that the scale is a unidimensional measure of nurse practice affordance, although, subscales such as nurse integration, efficiency, and/or direct patient care, may be evident in larger and more diverse samples. This is an area for future testing and development, including a confirmatory factor study to test the goodness-of-fit of one or multiple factors.

Based on the scope and timing of this study, development of the HDR CARE Scale Inpatient Version was focused on nurses who work on inpatient hospital units. Expanding upon this work, it will be possible to further refine and test related instruments for use in other healthcare settings, such as ambulatory care. This future step would increase the flexibility and adaptability of the instrument and expand its use to more studies of healthcare built environments.

As a tool specifically intended for healthcare design research to quantitatively measure nurses’ experience working in the care environment, the CARE Scale will be valuable in generating quantitative and standardized data, making possible measurement of associations between other factors, such as organizational support and procedures, and comparisons across time and facilities. Within the healthcare design industry, this capability can lead to greater knowledge about the aspects of a healthcare facility that best support nurses’ work and that are associated with their ability to provide quality patient care. The development of this method to capture this information quantitatively is vital to future research that begins to address causal pathways between the healthcare built environment and human outcomes.

## Supporting information

S1 Dataset Phase 1 pilot survey data. (XLSX)

S2 Dataset Phase 2 content validity survey data. (XLSX)

S3 Dataset Phase 3 cognitive interviews survey data. (XLSX)

S4 Dataset Phase 3 cognitive interviews coding summary by node. (XLSX)

S5 Dataset Phase 3 cognitive interviews content node count. (XLSX)

S6 Dataset Phase 4 pilot survey data. (XLSX)
